# The Antecedents and Consequences of Health Care Professional–Patient Online Interactions: Systematic Review

**DOI:** 10.2196/13940

**Published:** 2019-09-25

**Authors:** Lili Shang, Meiyun Zuo, Dan Ma, Qinjun Yu

**Affiliations:** 1 Research Institute of Smart Senior Care School of Information Renmin University of China Beijing China; 2 School of Basic Medicine Peking University Health Science Center Beijing China

**Keywords:** health care professional–patient interaction, online health care service, online communication, eHealth, review

## Abstract

**Background:**

Online health care services effectively supplement traditional medical treatment. The development of online health care services depends on sustained interactions between health care professionals (HCPs) and patients. Therefore, it is necessary to understand the demands and gains of health care stakeholders in HCP-patient online interactions and determine an agenda for future work.

**Objective:**

This study aims to present a systematic review of the antecedents and consequences of HCP-patient online interactions. It seeks to reach a better understanding of why HCPs and patients are willing to interact with each other online and what the consequences of HCP-patient online interactions are for health care stakeholders. Based on this, we intend to identify the gaps in existing studies and make recommendations for future research.

**Methods:**

In accordance with the PRISMA (Preferred Reporting Items for Systematic Reviews and Meta-Analyses) guidelines, a systematic retrieval was carried out from the Web of Science, PubMed, and Scopus electronic databases. The search results were confined to those papers published in English between January 1, 2000 and June 30, 2018. Selected studies were then evaluated for quality; studies that did not meet quality criteria were excluded from further analysis. Findings of the reviewed studies related to our research questions were extracted and synthesized through inductive thematic analysis.

**Results:**

A total of 8440 records were found after the initial search, 28 papers of which were selected for analysis. Accessibility to HCPs, self-management, and unmet needs were the main triggers for patients to participate in online interaction. For HCPs, patient education, career needs, and self-promotion were the major reasons why they took the online approach. There were several aspects of the consequences of HCP-patient online interactions on health care stakeholders. Consequences for patients included patient empowerment, health promotion, and acquisition of uncertain answers. Consequences for HCPs included social and economic returns, lack of control over their role, and gaining more appointments. HCP-patient online interactions also improved communication efficiency in offline settings and helped managers of online health care settings get a better understanding of patients’ needs. Health care stakeholders have also encountered ethical and legal issues during online interaction.

**Conclusions:**

Through a systematic review, we sought out the antecedents and consequences of HCP-patient online interactions to understand the triggers for HCPs and patients to participate and the consequences of participating. Potential future research topics are the influences on the chain of online interaction, specifications and principles of privacy design within online health care settings, and roles that sociodemographic and psychological characteristics play. Longitudinal studies and the adoption of text-mining method are worth encouraging. This paper is expected to contribute to the sustained progress of online health care settings.

## Introduction

Health care is closely related to people’s life. Previously, interactions between health care professionals (HCPs) and patients occurred mainly in physical hospital settings. Today, eHealth has transformed the pattern of health care delivery with the development of information and communication technology [1]. Online interactions between HCPs and patients are increasingly playing a role in the provision of health care services. As a result, many patients have become dual-path inquirers (online consultation and offline physical access), and HCPs have become dual-path service providers (online and offline). An HCP-patient online interaction here refers to the exchange of health-related information via the internet [2] between HCPs and patients without in-person, face-to-face contact. In the process of interaction, patients can ask questions about health-related matters and HCPs help them by delivering health care advice and support through an online channel [3,4].

With the rise of the internet, online channels for HCPs to interact with patients have gradually emerged, including email, internet portals, social media, and online health communities. Based on the mentioned interactive channels, two forms of online health care interaction are classified, namely written communication and oral communication [5,6]. In the case of online written communication, the interaction between patients and HCPs is based on text messages and does not require the use of interactive instruments concurrently [7]. For online oral communication, it is voice-based and characterized by continuous interaction, allowing professionals and patients to interact uninterruptedly over a period of time. Driven by managers, online health care settings that combine different forms and channels are constructed at a cost-benefit tradeoff to facilitate HCP-patient interaction. Managers also function as decision makers in health care settings, such as formulating patterns of service provision, measuring investment, pricing, and so on. There are three main stakeholders in online health care settings, including normal users (ie, patients or their advocates, collectively called *patients* in this paper), who seek and obtain health care advice; doctors who deliver health care advice and support (*HCPs* in this paper); and managers who make decisions on the operation and development of online health care settings [8].

There is an increasing number of studies referring to HCP-patient online interactions from the perspective of patients or HCPs, such as what triggers patients to consult previously unknown doctors online [9], what determine doctors’ reasons for the engagement in online interactions with patients [10], and what consequences the use of online channels has for participants [11]. The advancement of online health care settings is closely associated with sustained interaction between HCPs and patients [12], which is a manifestation of the socialization that indicates the activity level between members [13]. Thus, to define specific action items that help obtain an increased activity level, it is critical to identify what triggers the involvement of patients and HCPs in online interaction. Furthermore, the aim of providing services in the form of online interaction is primarily to improve health care [14]. At present, it engenders some consequences, including pros and cons that need to be further discussed and analyzed. To provide inspiration for the sustainable operation and effectiveness of online health care settings, it is necessary to examine this field thoroughly. Hence, this paper exhibits a systematic review of the antecedents and consequences of HCP-patient online interaction, and tries to answer the following research question: what are the antecedents and consequences of HCP-patient online interactions covered in the literature so far?

Ball and Lillis [1] conducted a literature review on the influence of eHealth on the HCP-patient relationship in 2001 before the rise of Web 2.0, which needs to be updated. After that, reviews related to our study, such as the impact of electronic communication on health care service provision [15], doctors’ professional use of the internet and factors that encourage their usage [10], and the effects of social media use [11,16], all focus on specific aspects of this topic. To date, there has been no other comprehensive literature review of the antecedents and consequences of HCP-patient online interaction. It is necessary to re-examine recent studies and conduct a more thorough review. In this paper, we try to identify the antecedents and consequences of HCP-patient online interaction. The antecedent and consequence factors identified in this study could be applied to expound the facilitators and outcomes involved in HCP-patient online interaction. Therefore, we intend to identify the gaps in existing studies and make recommendations for future research. This review is also expected to contribute to the sustained progress of online health care settings.

## Methods

### Retrieval Strategy

In accordance with the Preferred Reporting Items for Systematic Reviews and Meta-analyses (PRISMA) guidelines [17], a systematic retrieval was carried out including the retrieval of the electronic databases Web of Science, PubMed, and Scopus. An additional manual retrieval was performed on the search of references and the identification of studies that may have been missed, as confirmed by experts.

The search terms included all possible keyword combinations from three aspects of population, channels for online interaction, and health care settings. The population covers both sides involved in online interaction, including patients and HCPs, and possible channels for online interaction in all types of health care settings were taken into account. The search terms were limited to those commonly used (see Multimedia Appendix 1 for search strings by each database). The search results were confined to those articles published in English between January 1, 2000 and June 30, 2018, because the earlier studies were mainly exploratory tests [15]. Journal papers and conference papers were retained, and previous reviews relevant to this study were also accepted into our study for their reference value. All study designs were included to find more comprehensive evidence to address our research question.

### Selection of the Studies

The selection strategy was analyzed by the research team to reduce the possibility of bias. Two reviewers (LS and DM) worked together to conduct a comprehensive search and then excluded irrelevant literature. For those studies that they were not sure whether to exclude, two reviewers (MZ and QL) were assigned to deliberate it until they reached an agreement. To find the best evidence to address our research question, we defined explicit selection criteria for the inclusion of papers. Papers were included in this review if they studied (1) interactions between HCPs and patients via the internet and (2) the antecedents or consequences of HCP-patient online interaction.

According to the first criterion, the included papers needed to focus on online interaction, namely the bilateral exchange of health-related information via the internet. Therefore, studies focusing on unilateral information acquisition were excluded, such as the usage of physician-rating websites, the search for health information using search engines, and so on. In addition, the two roles of interaction had to be HCPs and patients. Thus, studies addressing peer-peer interaction, such as those just between patients or between HCPs, were not included in this study.

The second criterion indicates that any research that did not involve antecedents or consequences would be excluded, such as only comparing online interactions with offline interactions or focusing on data analysis methods of online interaction. Antecedents here refer to the factors that trigger patients and HCPs to participate in online interaction, whereas the factors impeding patients and HCPs to interact online were not taken into account. Consequences refer to the results brought on by online channel usage to health care stakeholders, including the benefits, risks, opportunities, challenges, and so forth.

### Data Extraction, Evaluation, and Analysis

To ensure the reliability of data and analysis, the work in this part was carried out by two reviewers (LS and DM) independently; the inconsistencies were solved by a third reviewer (MZ). We created an information form for selected literature to aggregate relevant research data. Similar to the existing study [10], published information on studies, including the aim of the study, channels for online health care interaction, study design, and characteristics of respondents, were collected as general information.

We used the Critical Appraisal Skills Programme quality assessment tool [18], which consists of 10 items, to evaluate qualitative research and studies using mixed methods. Three broad themes needed to be considered when appraising, including the statement of findings, the validity, and contribution of the research. The assessment tools for quantitative studies in this review were adapted from the US National Heart, Lung, and Blood Institute quality assessment tool [19] and criteria for the evaluation of quantitative research proposed by Tan and Goonawardene [20]. The criteria consist of 14 items and are used to check the clarity of objectives, the selection of samples and methods, the reliability of results, and the outcomes of the research. Moreover, review papers published in peer-reviewed journals were determined to be qualified due to the more rigorous methodology compared with those published without peer review [21]. Finally, studies that did not meet these quality criteria were excluded from further analysis.

We extracted and synthesized findings of the reviewed studies based on our research question related to the antecedents and consequences of HCP-patient online interaction. The data analysis procedure was divided into three stages. In the first stage, all the antecedent and consequence factors in the studies that clearly answered our research question were identified and listed. In the second stage, for those selected studies that did not directly answer our research question, we studied the findings saved in our database to conduct an inductive thematic analysis [22]. Thematic analysis enabled us to synthesize research findings in a transparent manner and facilitate the emergence of new concepts. We coded the text to capture its meaning and grouped them to form descriptive themes by comparing similarities and differences between codes. Then, a synthesis of results that addressed our research question emerged by generating new interpretations based on descriptive themes. In the last stage, antecedent or consequent factors with similar meanings identified in the first two stages formed a synthesis by induction.

## Results

### Overview

**Figure 1:**
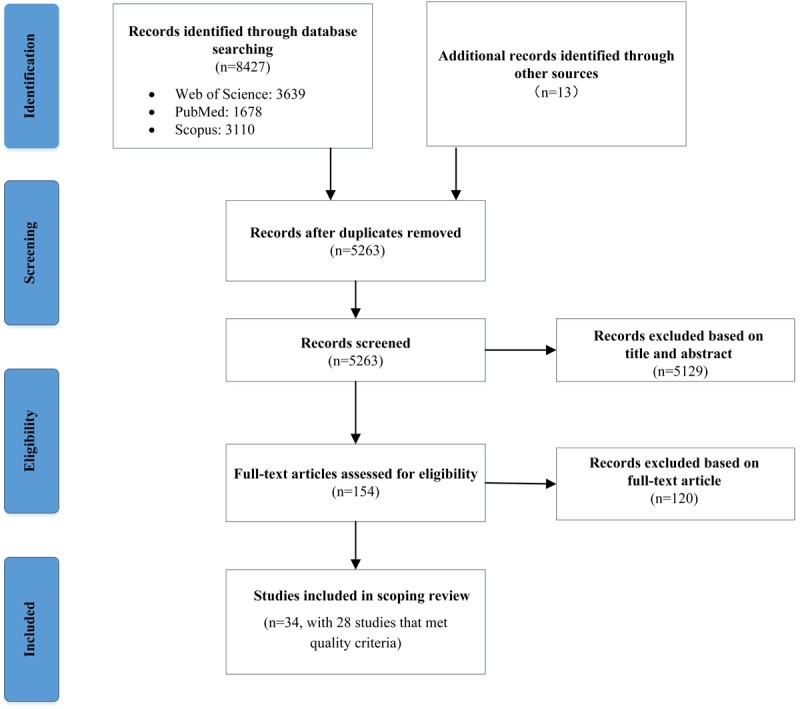
Flow diagram for the selection of literature.

A total of 8440 records were found after the initial search. All records were exported and sorted, and all duplicates from diverse sources were removed, which reduced the number of records to 5263. Another 5129 papers were excluded after screening titles and abstracts. Of the remaining 154 full-text articles assessed for eligibility, 120 were excluded. Therefore, 34 studies were included in the scoping review. Of the 34 selected studies, 6 were excluded after quality assessment [2,4,16,23-25]. Finally, 28 studies met the quality criteria and were included in the review. Figure 1 is a flow diagram that depicts the selection process and results at each stage. A list of selected studies in chronological order of publication is shown in Multimedia Appendix 2. For the quality assessment of the included studies, see Multimedia Appendix 3.

### Characteristics of the Reviewed Studies

Among the 28 included studies, 27 were journal papers and 1 was a conference paper. Research methods in the selected literature were quantitative study (n=15), qualitative study (n=8), mixed methods (n=1), and literature review (n=4). Channels for HCP-patient online interaction used in the targeted literature included email (n=3), online health community (n=14), social media (n=6), internet portal (n=4), desktop videoconferencing equipment (n=1), and other unspecified apps or health service websites (n=6). In most of these studies (n=27), HCPs interacted with patients mainly through written communication based on text messages. Only two studies involved online oral communication. Of the 28 articles, 7 explored the antecedents of online interaction, 11 discussed the consequences, and the rest studied both topics.

**Figure 2:**
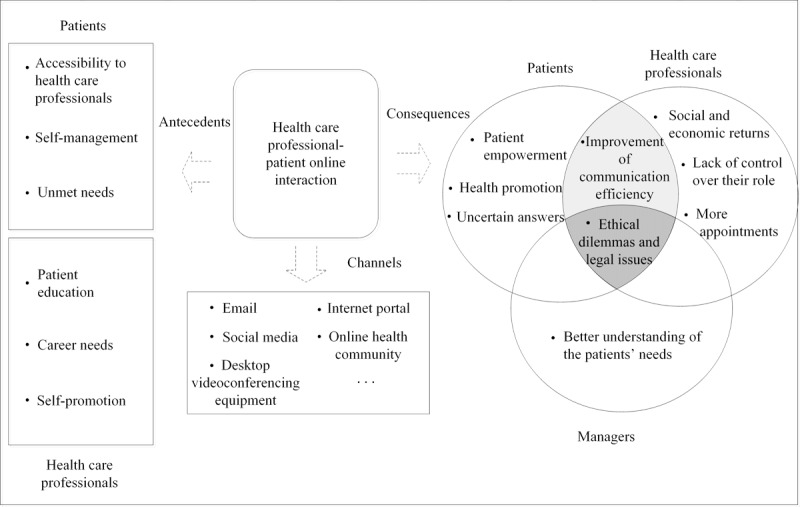
A visualization of antecedents and consequences of health care professional–patient online interaction.

The framework constructed from the emerging themes included (1) antecedents of online interactions between patients and HCPs and (2) consequences of online interactions on different health care stakeholders in general. For the antecedents of online interactions between patients and HCPs, we identified themes of antecedents from the perspective of two sides of online interaction: patients and HCPs. For the consequences of online interactions to health care stakeholders, we identified themes related to consequences of online interactions on three sides: patients, HCPs, and managers. Figure 2 is a visualization of the antecedents and consequences of HCP-patient online interactions identified in this paper.

### Antecedents of Health Care Professional–Patient Online Interaction

Selected literature examined the factors that triggered patients and HCPs to participate in online interaction. A summary of these antecedents in descending order by the total number of related literature is shown in Table 1.

### Antecedents From the Perspective of Patients

The reviewed studies showed that accessibility to HCPs, self-management, and unmet needs are the main factors that trigger patients to participate in online interaction.

**Table 1:** Summary of the antecedents of health care professional–patient online interactions from the perspectives of patients and health care professionals.

Perspectives and antecedents		Description	Number of studies
**Patients**			
	Accessibility to health care professionals	Availability for patients to access to health care professionals remotely at any time; support for anonymous access	5
	Self-management	Primary evaluation of a medical problem before an offline appointment; preparing for future consultations in physical settings; a more effective way of health conditions self-management	5
	Unmet needs	Discontent with health care previously received in offline settings; more alternative choices of health care services than offline settings	5
**Health care professionals**			
	Patient education	An effective way to educate patients, especially patients with chronic diseases, about behavioral changes and drug adherence; a well-suited approach to deepen patients’ awareness of a specific health condition	8
	Career needs	An essential way to provide health care services to patients in the digital age	5
	Self-promotion	Broader self-promotion to others to increase reputation and popularity	3

For many patients in the included studies, a major reason for choosing online consultation was that they had easier access to HCPs. The online channel offers a venue for patients to access doctors—even prestigious doctors—remotely [15] and discuss sensitive issues anonymously [26] at any time [9,27], where patients do not have to wait too long and can save money from not going to physical settings repeatedly [1]. In addition, the need for effective self-management of their conditions drove patients to make an online consultation. Patients, especially those with long-term conditions (eg, patients with diabetes), thought they should be responsible for their condition management, so they independently sought solutions and gained power to manage their own situations [28,29], such as preventive care [15], preparation for future consultations [9], and future treatment trajectory [30] in physical hospitals. Furthermore, the unmet needs of patients through offline channels were essential in determining their online counseling as a source of support. Patients expressed discontent with the health care they previously received offline [9,31] (eg, inadequate information, lack of trust, and hard to understand); therefore, they needed a second opinion from online HCPs [1,26,30].

### Antecedents From the Perspective of Health Care Professionals

For HCPs, patient education, career needs, and self-promotion were found to be the main factors that triggered them to participate in online interaction. More specifically, professionals saw patient education as a major driver of the use of online channels as tools [1,27,32-35,37], such as encouraging health behavioral changes and drug adherence, raising public awareness about specific health conditions, and eliminating misunderstandings about certain diseases. Moreover, career needs acted as a trigger for HCPs to provide online health care services [10,32,36-37], which in return provided them with opportunities for future career development. The motivation of self-promotion was also a relatively important reason for participating in online interactions [32,37,38]. For HCPs, they wanted to present themselves to colleagues and patients through online interaction, and they considered it helpful to obtain better career development at their institutions.

### Consequences of Health Care Professional–Patient Online Interactions for the Different Health Care Stakeholders

The selected literature indicated that HCP-patient online interactions affected online health care stakeholders, including patients, HCPs, and managers. A summary of these consequences in descending order according to the total number of related literature is shown in Table 2.

**Table 2:** Summary of the consequences of health care professional–patient online interactions on different health care stakeholders in general.

Stakeholders and consequences		Description	Number of studies
**Patients**			
	Patient empowerment	More empowerment for patients	7
	Health promotion	Improvement of the patients’ health conditions; increase of patient adherence to treatments	4
	Uncertain answers	Unavailability of clear answers due to insufficient patient information held by health care professionals	2
**Health care professionals**			
	Social and economic returns	Acquisition of social returns, such as better reputation and greater popularity; acquisition of economic returns, such as online votes, bonus, likes, and electronic gifts from patients	4
	Lack of control over their role	Leading to a lack of private time and life in disorder; service requests beyond one’s professional capacity	4
	More appointments	Potential opportunities to have more patient appointments	3
**Managers**			
	Better understanding of the patients’ needs	Useful insights for online health care service managers to understand the patients’ needs, especially in terms of service delivery and pricing strategies	4
**Patients, health care professionals**			
	Improvement of communication efficiency in offline settings	Improved ability of patients describing a specific health problem; increased face-to-face communication skills of professionals on account of the experience of formulating online text-based answers	3
**All stakeholders**			
	Ethical dilemmas and legal issues	Unauthorized dissemination of personal information of health care professionals by patients; professional’s uncertainty about the legitimacy whether or not to use online public information about patients and the rationality of online private communication with patients; ambiguity of information authorization for managers	9

### Consequences of Online Interactions for Patients

Consequences of HCP-patient online interactions for patients included patient empowerment, health promotion, and uncertain answers. HCP-patient online interactions contributed to patient empowerment [1,26-28,34,39,40]. Online interactions seemed to operate in a different paradigm from offline communication because the professional was no longer at first place in the sequence and the problem was initiated by the patient, ultimately forming a patient-centered pattern [40]. Patients gained a variety of empowerment outcomes after they were provided with online health care service, such as the reinforced capacity to manage their physical conditions and increased acceptance of the disease, enhanced self-efficacy, and it promoted quality of interactions with professionals.

Studies demonstrated the role of HCP-patient online interactions in driving health promotion behaviors of patients, such as ending bad habits, developing a regular schedule, keeping a balanced diet, increasing physical activities, and maintaining a healthy mental state [36,34,41,42]. In particular, online health care interactions can effectually improve patient adherence, such as reminding patients of punctual intake of prescribed medicine, thus contributing to a successful treatment.

In an online health care environment, patients occasionally receive uncertain answers [26,39]. Due to insufficient patient information through online interaction, HCPs find it difficult to provide precise answers to patients and often advise them to make an appointment with an offline doctor. Furthermore, the information provided to professionals who need to respond is mainly text-based, and they seldom have the option to perform medical checks [26]. Therefore, assessing the adequacy or even veracity of the text-based information provided by patients may be impractical [39].

### Consequences of Online Interactions for Health Care Professionals

Consequences for HCPs include social and economic returns, lack of control of their role, and gaining more appointments. The participating professionals gained returns from online channels [3,6,33,43]. Two notable ones for participating HCPs were social and economic returns. Specifically, by interacting with patients online, HCPs may obtain social returns such as increased reputation and popularity, and economic returns such as online votes, bonus, likes, and electronic gifts from patients [3].

Studies identified that HCP-patient interactions led to professionals feeling a lack of control of their role [11,15,27,37]. For example, Atanasova et al [37], in an in-depth semistructured interview, found all HCPs in online health communities reported experiences of overload and high burden. Professionals also experienced overcommitment to online interaction. They expressed that the demands of patients were beyond their capacity in the online context, and they were skeptical of the demand-and-supply patterns triggered by these health-related technological innovations [37].

Health care professional–patient online interactions provided professionals with opportunities to get more appointments [6,33,35]. Professionals felt that interactions in online settings enabled them to receive more patients. In addition, studies showed that HCP-patient online interactions could help professionals, especially those with low titles, get more appointments through accumulating more online experience [33].

### Consequences of Online Interactions for Managers

Online HCP-patient interactions help managers better understand the needs of patients. Online health care interaction channels have accumulated rich data reflecting the trajectory of users’ behavior. The included studies support evidence that data analysis helps managers understand patient needs [3,34,44,45]. For example, Yang et al [44] studied the influence of the process of delivering online services on patient satisfaction with professionals. Results showed that the response speed of professionals and the interaction frequency between professionals and patients—two important variables in the process of delivering online services—positively affected patient satisfaction. Wu and Lu [45] investigated the influence of online service provision and pricing on patient satisfaction. The results indicated HCPs who provide more services have higher patient satisfaction, and the relationship between service price and patient satisfaction is an inverted U shape. The price difference between different services provided by HCPs significantly decreases patient satisfaction.

### Other Consequences of Online Interactions for Health Care Stakeholders

Health care professional–patient online interactions can also have an effect on the improvement of communication efficiency in offline settings [26,34,39]. Online interaction, normally used as a basis for further offline discussions with doctors, can help patients better understand and express their health status, problems, treatments, and remedies [34,39]. Developing responses to text-based health care consultations also promotes face-to-face communication skills for professionals [26]. With online interactive experience, they learn more about how to formulate medical terms clearly and how to properly refer to internet resources to provide a wide range of medical information [26].

In the context of online health care interaction, all health care stakeholders have encountered ethical dilemmas and legal issues [11,26,27,32,36-35,46]. For patients, in addition to giving online appraisals to professionals, they may also spread unauthorized personal information about professionals on the internet [46]. For HCPs, they are confused about how to respond to online ethical dilemmas [37]. Moreover, there is no general agreement on the appropriateness of using patient’s publicly available online information (eg, patients’ private life updates on their Facebook pages) to assist them in treatment. Professionals also argue that the separation of their professional and private lives should be considered. Whether private communication between professionals and patients online should be encouraged remains to be seen. The most common way that professionals deal with undesired private communication is to change the privacy settings of their online interactive apps, followed by ignoring a friend request from their patients [35]. For managers, they are bound to face the threat posed by the disclosure of private information when empowering patients to deliver information about professionals to public [46]. In some cases, profiles of HCPs have been developed and displayed on third-party review sites without their personal participation and confirmation.

## Discussion

### Principal Findings

This study presents the antecedents and consequences of HCP-patient online interactions covered in the existing literature. In the reviewed studies, the most reported channels of online professional–patient interactions were online health communities and social media, especially in recent years. Interactions between HCPs and patients were based mainly on written communication, whereas a few studies were based on oral communication. The findings of this study provide evidence that the unique advantages of online health care settings over offline ones drive patients and HCPs to participate in online interaction. In addition, HCP-patient online interactions do have positive effects on patients’ health care, although it also has limitations. For managers, it provides opportunities and poses challenges for them.

Antecedents that trigger patients to participate in online interactions were identified. The findings of this study show that accessibility to HCPs, self-management, and unmet needs in offline channels are the main factors that trigger patients to participate in online interaction, echoing the views of previous reviews [1,15]. These factors also imply the demands of patients for health care services and have the potential to provide insights into how to design online health settings to meet patients’ needs. For example, it is necessary to attract more well-known doctors who are difficult to make an appointment with in offline settings to provide online health care services so that patients can have access to them. Also, managers of online settings could consider defining functions and processes that help patients, especially those with chronic conditions, perform self-management.

Antecedents that encourage HCPs to interact online include both altruistic factors (patient education), which have been determined in an existing review [1], and egoistic factors (career needs and self-promotion), which have been updated in this study. When it comes to altruistic factors, the use of an online channel can be a favorable way to provide patient education for HCPs. Egoistic factors include career needs and self-promotion. Consistent with previous findings [10], HCPs consider the use of online channels as a part of their career development. The newly identified antecedent, self-promotion, suggests HCPs want to take advantage of online approaches to present themselves to colleagues and patients to help their future. Given this, it is necessary for online health care settings to consider incorporating functions or items that facilitate self-presentation of HCPs.

Compared with the consequences of online interactions for patients and HCPs presented in previous reviews, this paper found new positive and negative effects. Positive effects for patients include patient empowerment and health promotion. However, uncertain answers from online HCPs, as a passive consequence, imply patients are often dissatisfied with the vague suggestions from online HCPs, and then revisit their private doctors. The positive consequences of online interactions identified in reviewed studies for HCPs are social and economic returns and more appointments from patients. The negative side is that HCPs feel a lack of control over their role because the appointments and interactions with patients remotely can occur at any time. The improvement of communication efficiency in offline settings was found to have a positive impact on online interactions for both patients and HCPs. In general, compared with previous reviews, the newly identified consequence factors in this review include uncertain answers, social and economic returns, more appointments, and the improvement of communication efficiency in offline settings. Further, we found more positive evidence of the consequences of online interactions than negative ones.

The consequences of online HCP-patient interactions for managers were also examined in the reviewed studies. Specifically, in the process of online interaction, a large amount of potentially useful data are produced, providing opportunities for managers to have a better understanding of patients’ needs. Meanwhile, ethical and legal issues are emerging, which pose challenges for managers. In the future, it will be necessary for managers to take advantage of opportunities and address the previously mentioned challenges to enable cost-benefit management.

### Limitations of the Review

To obtain more comprehensive evidence of antecedent and consequence factors, this review included diverse and heterogeneous studies with different research analyses and methods. The channels and forms of online interactions are changing, and the motivation of people to participate, and the consequences of participation, may also evolve. However, the heterogeneity of studies is a challenge to reveal the dynamics of the antecedent and consequence factors. If studies adopt a uniform format, comparisons and trends could be more accurately identified.

This review focuses on identifying the antecedents and consequences of online interactions separately, without centering on the influencing chain. It should be noted that factors impeding patients and HCPs from interacting online were not within the scope of our review; therefore, no salient themes were identified in the coding process of the included literature. It would be more comprehensive to incorporate these factors. The entire influencing chain from the antecedents to the online interaction (feelings, thoughts, problems, advantages, and disadvantages) and further on to the consequences would be interesting for a literature review.

### Recommendations for Future Studies

Recommendations for future research are from the aspects of potential topics and methods. There are three potential research topics deserving further exploration. First, there is a distinct lack of research on systematically investigating the influencing chain of online interactions that may explain how online health care settings work. It will be meaningful for future original research to uncover the influencing chain of online interaction.

Second, the specifications and principles of privacy design in the context of online health care settings should be considered in future research. With the emergence of online channels, the paradigm of interaction between HCPs and patients has changed, and the guidelines for interaction should also change [46]. However, professionals sometimes still rely on experiences and intuition to interact, and patients may be confused about how to express themselves effectively to get definite answers. Next, most studies have mentioned privacy considerations for health care stakeholders while using these technologies to communicate on social media [47], but only a few feasible solutions have been provided [48]. Consequently, it is necessary to probe the legal and ethical problems in current situations and explore specifications of consultation and service delivery.

Third, the role of sociodemographic and psychological characteristics in existing studies has been underestimated. Demand for online health care interaction of professionals or patients with different characteristics may vary. For example, this type of interaction might be favored by surgeons because of the more effective means of preoperative preparation and the short-term follow-up after surgery it provides [8,49]. In addition, patients with high-risk diseases show higher sensitivity to the process of online interactions [44], and elderly patients’ demands for online health care interactions may be different from that of young people [50]. Social presence, the degree to which a person is perceived as “real” and connected to others in the process of communicating through the media [51], and the health-related information-seeking personality, a need for cognition and information more than others when making decisions, may also influence the use of online interaction channels [52]. Therefore, future research could aim to explore how these characteristics play a role in this topic.

The existing studies can be extended from two aspects of research methods. First, more longitudinal studies on HCP-patient interactions should be performed in the future. Previous studies were mainly conducted by cross-sectional design to investigate the effect of online interactions on patients’ health conditions, without reflecting the dynamic effects of online interactions [53]. Therefore, long-term observations should be encouraged to investigate the dynamics of the consequences of online interaction. In addition, the text-mining method is worth encouraging to analyze text messages generated by online interaction. Through text mining, studies have characterized communication patterns in the process of health information seeking [54] and identified influential users in online health communities [55], yet there is a lack of analysis of the interactive content itself. Therefore, future research can attempt to carry out text analysis of the content generated in the course of interactions and seek out more precise antecedent or consequence factors of online interactions to help guide the design of more effective patient-centered online health care settings [56].

## Appendix

Multimedia Appendix 1 Search strings by each database.

Multimedia Appendix 2 Summary of selected literature.

Multimedia Appendix 3 Quality assessment of included studies.

## Abbreviations

HCP: health care professional
