# Supplementary material for: The Antecedents and Consequences of Health Care Professional–Patient Online Interactions: Systematic Review
Source: J Med Internet Res. 2019 Sep 25;21(9):e13940. doi: 10.2196/13940 (PMC6785718; doi:10.2196/13940)
Supplement: Multimedia Appendix 1 [file jmir_v21i9e13940_app1.pdf]

**Multimedia Appendix 1. Search strings by each database.**

Database: Web of Science
TI=((doctor* OR patient* OR physician* OR "healthcare professional")AND (internet OR online OR electronic OR web* OR "social media" OR "social network" OR "ICT" OR "OHC" OR "e-health") )AND TS=( health* OR medical OR "medicine")
Types of literature: ARTICLE OR MEETING
Databases= WOS
Time span =2000-2019
Retrieval language = automatic

Database: PubMed
(((doctor[Title] OR patient[Title] OR physician[Title] OR health professional[Title] OR healthcare professional[Title]) AND (Internet[Title] OR online[Title] OR web[Title] OR electronic[Title] OR social media[Title] OR social network[Title] OR ICT[Title] OR OHC[Title] OR e-health[Title]) AND (health [All Fields] OR medical[All Fields] OR medicine[All Fields])) AND ( "2000/01/01"[PDat] : "2018/06/30"[PDat] ))

Database: Scopus
( TITLE ( ( "doctor" OR "patient" OR "physician" OR "healthcare professional" ) AND ( "Internet" OR "online" OR "web" OR "electronic" OR "social media" OR "social network" OR "ICT" OR "OHC" OR "e-health" ) ) AND TITLE-ABS-KEY ( "health" OR "medical" OR "medicine" ) ) AND DOCTYPE ( ar OR re ) AND PUBYEA R > 1999 AND PUBYEAR < 2019 AND ( LIMIT-TO ( LANGUAGE , "English" ) )
