# Supplementary material for: The Antecedents and Consequences of Health Care Professional–Patient Online Interactions: Systematic Review
Source: J Med Internet Res. 2019 Sep 25;21(9):e13940. doi: 10.2196/13940 (PMC6785718; doi:10.2196/13940)
Supplement: Multimedia Appendix 2 [file jmir_v21i9e13940_app2.pdf]

Multimedia Appendix 2. Summary of selected literature (n=34).

ID[a]	Study	Objectives of the study	Method/ Design	Channels	Samples	Number of samples
**A01**	Ball MJ, Lillis J. E-health: transforming the physician/patient relationship[J]. International Journal of Medical Informatics, 2001 [1]	To present a picture of needs of patients for online interaction and how Internet facilitates the health delivery of doctors and organizations	Review	Internet portal and e-mail	/	/
**A02**	Umefjord G, Petersson G, Hamberg K. Reasons for consulting a doctor on the Internet: Web survey of users of an Ask the Doctor service[J]. Journal of Medical Internet Research, 2003 [9]	To study why individuals choose to consult previously-unknown doctors on the Internet	Mixed methods /Web survey	Internet portal of Infomedica: Ask the Doctor service	inquirers of 'Ask the Doctor' service	3,622 inquirers involved, 1,223 valid surveys
**A03**	McGeady D, Kujala J, Ilvonen K. The impact of patient–physician web messaging on healthcare service provision[J]. International Journal of Medical Informatics, 2008 [15]	To address what is the impact of electronic communication to the efficiency and effectiveness of health care service provision	Review	patient–physician web messaging sites and e-mail	/	/
**A04**	Masters K. For what purpose and reasons do doctors use the Internet: a systematic review[J]. International Journal of Medical Informatics, 2008 [10]	To examine doctors' using the Internet, and the factors that influence their usage	Review	Internet (unspecified) and email	/	/
**A05**	Dixon RF, Stahl JE. A randomized trial of virtual visits in a general medicine practice[J]. Journal of Telemedicine and Telecare, 2009 [29]	To examine the feasibility, effectiveness and acceptability of a face-to-face office visit compared to a virtual visit	Quantitative study /Randomized trial	desktop videoconferencing equipment: Polycom	patients from an adult primary care practice in Boston who were diagnosed with	175 patients involved, 152 valid trials

					hypertension, diabetes, hypercholesterolaemia or acute illnesses	
**A06**	Lee SY, Hawkins R. Why do patients seek an alternative channel? The effects of unmet needs on patients' health-related Internet use[J]. Journal of Health Communication, 2010 [31]	To understand what causes women with breast cancer to use online health tools to seek medical advice from an expert	Quantitative study /Randomized trial	an ehealth system: CHESS	women who were newly diagnosed with breast cancer, recruited from 1999 to 2002 from cancer centers	122 women with breast cancer
**A07**	Hewitt-Taylor J, Bond CS. What e-patients want from the doctor-patient relationship: content analysis of posts on discussion boards[J]. Journal of Medical Internet Research, 2012 [28]	To ascertain what people with diabetes who use Internet discussion forums want from their doctors	Qualitative study / Content analysis	4 Internet discussion boards	Two of the boards were owned by organizations from the United States and 2 were owned by organizations from the United Kingdom, but participants on these boards came from all over the world	All posts on Internet discussion boards for diabetes
**A08**	Daniel DS, Graham D, Thompson MA, Johnson, LJ, Johnston, C, Fisch, MJ, Miller R. Practical guidance: the use of social media in oncology practice[J]. Journal of Oncology Practice, 2012 [23]	To present issues about the use of social media channels for doctor-patient communication and provide specific guidance to individual practitioners on how to engage in social media	Qualitative study /Descriptive analysis	Any types of social media	/	/
**A09**	Farnan JM, Sulmasy LS, Worster BK, Chaudhry HJ, Rhyne JA, Arora VM. Online medical professionalism: patient and public	To analyze medical ethics and professionalism issues in online postings and interactions	Qualitative study /Descriptive	Any types of online channel	/	/

	relationships: policy statement from the American College of Physicians and the Federation of State Medical Boards[J]. Annals of Internal Medicine, 2013 [2]		analysis			
**A10**	Courtney K. The use of social media in healthcare: organizational, clinical, and patient perspectives[J]. Enabling Health and Healthcare Through ICT: Available, Tailored and Closer, 2013 [16]	To explore the impacts of social media on health care organizations, clinicians, and patients	Review	Any types of social media	/	/
**A11**	Gholami-Kordkheili F, Wild V, Strech D. The impact of social media on medical professionalism: a systematic qualitative review of challenges and opportunities[J]. Journal of Medical Internet Research, 2013 [11]	To present social media–related challenges to medical professionalism and social media–related opportunities to either undermine or improve medical professionalism	Review	Any types of social media	/	/
**A12**	George DR, Rovniak LS, Kraschnewski JL. Dangers and opportunities for social media in medicine[J]. Clinical Obstetrics and Gynecology, 2013 [24]	To summarize the dangers and opportunities for social media in medicine	Qualitative study /Descriptive analysis	Any types of social media	/	/
**A13**	Brown J, Ryan C, Harris A. How doctors view and use social media: a national survey[J]. Journal of Medical Internet Research, 2014 [46]	To examine the impact of the use of social media on physicians' knowledge, attitudes, skills, and behaviors in practice and the influence of concerns about ethics, professionalism, and privacy upon that use	Quantitative study / Web survey	Any types of social media	doctors who had email addresses on the Australasian Medical Publishing Company (AMPCo) database	1,500 doctors involved, 187 valid questionnaires

**A14**	Schook RM, Linssen C, Schramel FM, Festen J, Lammers E, Smit EF, Westerman MJ. Why do patients and caregivers seek answers from the Internet and online lung specialists? A qualitative study[J]. Journal of Medical Internet Research, 2014 [30]	To explore the reasons why patients with lung cancer search the Internet for information and ask online lung specialists questions on the DLIC's interactive page	Qualitative study /Semi-structured telephone interview	the Dutch Lung Cancer Information Center (DLIC) website	visitors who asked the online lung specialists a question on the interactive page of the DLIC website were invited	5 lung cancer patients and 20 caregivers
**A15**	Campbell BC, Craig CM. Social media and health: current and future healthcare provider perspectives[J]. J Contemp Med Edu, 2014 [32]	To examine motivations of health care students and health care professionals for utilizing social media sites, and to determine their concerns of SM usage	Quantitative study / Web survey	highly utilized social media, including Facebook, Twitter, LinkedIn, Google+ and YouTube	health care students and health care professionals from the Texas Tech University Health Sciences Center	4,370 health care students and 4,269 health care professionals involved, 367 valid surveys
**A16**	Liu X, Guo X, Wu H, Vogel D. Doctor's Effort Influence on Online Reputation and Popularity[C]//International Conference on Smart Health. Springer, Cham, 2014 [33]	To examine why doctors participate in online activities and how they fully use online effort	Quantitative study /econometrics	Online health community: Haodf.com	data collected from Haodf.com	673 doctors
**A17**	Ventola CL. Social media and health care professionals: benefits, risks, and best practices[J]. Pharmacy and Therapeutics, 2014 [25]	To explore the benefits, risks, and guidelines for medical professionals using social media	Qualitative study /Descriptive analysis	Any types of social media	/	/
**A18**	Haluza D, Jungwirth D. ICT and the future of health care: aspects of health promotion[J]. International Journal of Medical Informatics, 2015 [34]	To assess the prevailing opinions and expectations among Austrian stakeholders regarding ICT-assisted health promotion	Qualitative study/Online Delphi survey	Any types of Information and Communication Technologies (ICT) applications	experts representing health care professionals, patient advocates, and administrative personnel engaged in the Austrian	94 participants in the first round, 73 participants (31 health care

					health care sector	professionals, 21 patient advocates, 21 administrative personnel) in the second round
**A19**	Yang H, Guo X, Wu T. Exploring the influence of the online physician service delivery process on patient satisfaction[J]. Decision Support Systems, 2015 [44]	To investigate the effects of the online service delivery process on patient satisfaction	Quantitative study /Econometrics	Online health community: Haodf.com	data of physicians responding to patients' questions on Haodf.com	2,112 physicians' personal responses
**A20**	Haluza D, Jungwirth D. ICT and the future of health care: Aspects of doctor-patient communication[J]. International Journal of Technology Assessment in Health Care, 2014 [35]	To identify specifications and perceptions of different interest groups regarding future demands of ICT-supported doctor–patient communication in Austria	Qualitative study /Online Delphi survey	Any type of Information and Communication Technology (ICT) applications	experts representing health care professionals, patient advocates, and administrative personnel engaged in the Austrian health care sector	94 participants in the first round, 73 participants (31 health care professionals, 21 patient advocates, 21 administrative personnel) in the second round
**A21**	Zummo ML. Exploring web-mediated communication: A genre-based linguistic study for new patterns of doctor-patient	To analyze how doctor–patient communication is affected by the medium and how health professionals	Qualitative study	health service websites including netdoctor.co.uk,	disease-related posts (cancer, migraine, diabetes, skin problems) collected	500 users' questions and 505 medical

	interaction in online environment[J]. Communication & Medicine, 2015 [39]	overcome issues concerning the virtual medical visit		doctorslounge.com, drjoshua.com, askmedicaldoctor.net and askadoctor.com	from five websites	answers
**A22**	Guo X, Guo S, Vogel D, Li Y. (2016). Online healthcare community interaction dynamics[J]. Journal of Management Science and Engineering, 2016 [4]	To investigate doctor-patient interaction dynamics from the dual perspectives of doctors and patients	Qualitative study	Any types of online health community	/	/
**A23**	Wu H, Lu N. Online written consultation, telephone consultation and offline appointment: An examination of the channel effect in online health communities[J]. International Journal of Medical Informatics, 2017 [6]	To examine how online channel usage affects offline channels and how the channel effects change with doctors' online and offline reputation	Quantitative study /Econometrics	Online health community: Haodf.com	web pages of doctors and information about doctors from Haodf.com	4254 web pages of doctors
**A24**	Björk AB, Hillborg H, Augutis M, Umefjord G. Evolving techniques in text-based medical consultation–Physicians' long-term experiences at an Ask the doctor service[J]. International Journal of Medical Informatics, 2017 [26]	To investigate an appropriate text-based medical consultation by learning from long-term experiences	Qualitative study/Semi-structured telephone interview	Health portal:1177 Vårdguiden	health care professionals that had the most long-term experience of the answering service of 1177 Vårdguiden.se	12 HCPs involved, 10 valid interviews
**A25**	Atanasova S, Kamin T, Petrič G. Exploring the benefits and challenges of health professionals' participation in online health communities: Emergence of (dis) empowerment processes and outcomes[J].	To explore the main benefits and challenges of health professional moderators' participation in the OHCs	Qualitative study/Semi-structured interview	Online health community: Med.Over.Net	health professional moderators from Med.Over.Net	7 HCPs

	International Journal of Medical Informatics, 2017 [36]					
**A26**	Zhang X, Liu S, Deng Z, Chen X. Knowledge sharing motivations in online health communities: A comparative study of health professionals and normal users[J]. Computers in Human Behavior, 2017 [38]	To explore motivations on health knowledge sharing of health professionals and normal users in online health communities	Quantitative study/ Web survey	Online health communities including haodf.com, babytree.com and dxy.cn	health professionals or normal users with experience in OHCs	A total of 627 responses, 443 valid responses
**A27**	Petrič G, Atanasova S, Kamin T. Impact of social processes in online health communities on patient empowerment in relationship with the physician: emergence of functional and dysfunctional empowerment[J]. Journal of Medical Internet Research, 2017 [40]	To examine the impact of social processes in OHCs on functional and dysfunctional patient empowerment in relationship with the physician	Quantitative study/Web survey	Online health community: Med.Over.Net	registered users of Med.Over.Net, the most popular general OHC in Slovenia	2,587 respondents involved, 1,572 valid surveys
**A28**	Guo S, Guo X, Fang Y, Vogel D. How doctors gain social and economic returns in online health-care communities: a professional capital perspective[J]. Journal of Management Information Systems, 2017 [3]	To examine the effects of status capital and decisional capital on doctors' social and economic returns in online health communities	Quantitative study /Econometrics	Online health community: Haodf.com	web pages of doctors and information about doctors from Haodf.com	39,911 doctors' information
**A29**	Wu T, Deng Z, Feng Z, Gaskin DJ, Zhang D, Wang R. The Effect of Doctor-Consumer Interaction on Social Media on Consumers' Health Behaviors: Cross-Sectional Study[J]. Journal of Medical Internet	To investigate how doctor-consumer interaction in social media affects consumers' health behaviors	Quantitative study / Web survey	Any types of social media	social media consumers who had experienced interactions with doctors on social media	A total of 435 responses, 352 valid responses

	Research, 2018 [41]					
**A30**	Wu T, Deng Z, Zhang D, Buchanan PR, Zha D, Wang R. Seeking and using intention of health information from doctors in social media: The effect of doctor-consumer interaction[J]. International Journal of Medical Informatics, 2018 [42]	To investigate how doctor-consumer interaction in social media influences consumers' health information seeking and usage intention	Quantitative study / Web survey	Any types of social media	social media consumers who had experienced interactions with doctors on social media	A total of 435 responses, 352 valid responses
**A31**	Daniel F, Jabak S, Sasso R, Chamoun Y, Tamim H. Patient-Physician Communication in the Era of Mobile Phones and Social Media Apps: Cross-Sectional Observational Study on Lebanese Physicians' Perceptions and Attitudes[J]. JMIR Medical Informatics, 2018 [37]	To describe the views of Lebanese physicians toward the use of social media and other online apps as means of patient communication	Quantitative study / Web survey	Social media including WhatsApp, Facebook, LinkedIn and Twitter	Practicing and training physicians from all medical specialities at American University of Beirut Medical Center (AUBMC) with an email address in the AUBMC directory	270 physicians involved, 238 valid surveys
**A32**	Atanasova S, Kamin T, Petrič G. The benefits and challenges of online professional-patient interaction: Comparing views between users and health professional moderators in an online health community[J]. Computers in Human Behavior, 2018 [27]	To explore and compare the benefits and challenges of online professional-patient interactions for users and health professional moderators and the effects on face-to-face medical encounters	Qualitative study/Semi-structured interview	Online health community: Med.Over.Net	Normal users and health professional moderators from Med.Over.Net	8 normal users and 7 health professional moderators
**A33**	Wu H, Lu N. Service provision, pricing, and patient satisfaction in online health communities[J]. International Journal of Medical Informatics, 2018 [45]	To empirically investigate the effects of service provision, pricing and price difference on patient satisfaction	Quantitative study /Econometrics	Online health community: Haodf.com	web pages of doctors and information about doctors from Haodf.com	2,309 doctors' information

**A34**	Guo S, Guo X, Zhang X, Vogel D. Doctor–patient relationship strength's impact in an online healthcare community[J]. Information Technology for Development, 2018 [43]	To examine the strength of the D– P relationship and its impacts on physicians' individual outcomes in online health communities	Quantitative study /Structural equation modeling analysis	Online health community: Haodf.com	web pages of doctors and information about doctors from Haodf.com	1,430 doctors' information

a: The literature A05 and A33 involve online oral communication.

In addition to A05, all of the literature involves online written communication.
