# Supplementary material for: The Antecedents and Consequences of Health Care Professional–Patient Online Interactions: Systematic Review
Source: J Med Internet Res. 2019 Sep 25;21(9):e13940. doi: 10.2196/13940 (PMC6785718; doi:10.2196/13940)
Supplement: Multimedia Appendix 3 [file jmir_v21i9e13940_app3.pdf]

Multimedia Appendix 3. Quality assessment of included studies.

Part 1. Quality assessment for qualitative and mixed-methods studies(n=14)[a]

The Quality Assessment Tool
(1) Was there a clear statement of the aims of the research?
(2) Is a qualitative methodology appropriate?
(3) Was the research design appropriate to address the aims of the research?
(4) Was the recruitment strategy appropriate to the aims of the research?
(5) Was the data collected in a way that addressed the research issue?
(6) Has the relationship between researcher and participants been adequately considered?
(7) Have ethical issues been taken into consideration?
(8) Was the data analysis sufficiently rigorous?
(9) Is there a clear statement of findings?
(10) Did the researcher discuss the contribution of the study to existing knowledge or understanding?

ID	First author	(1)	(2)	(3)	(4)	(5)	(6)	(7)	(8)	(9)	(10)	Qualified or not?
A02	Umefjord et al. [9]	1	1	1	1	1	0	1	1	1	1	Yes
A07	Hewitt-Taylor et al. [28]	1	1	1	1	1	0	1	1	1	1	Yes
A08	Dizon et al. [23]	1	2	2	2	2	2	2	2	0	0	No
A09	Farnan et	1	1	1	2	2	2	2	2	1	1	No

	al. [2]											
A12	George et al. [24]	1	2	2	2	2	2	2	2	1	1	No
A14	Schook et al.[30]	1	1	1	1	1	1	1	1	1	1	Yes
A17	Ventola [25]	1	2	2	2	2	2	2	2	1	1	No
A18	Haluza et al.[34]	1	1	1	1	1	0	0	1	1	1	Yes
A20	Haluza et al. [35]	1	1	1	1	1	0	1	1	1	1	Yes
A21	Zummo [39]	1	1	1	1	1	1	1	1	1	1	Yes
A22	Guo et al. [4]	1	2	2	2	2	2	2	2	1	1	No
A24	Björk et al. [26]	1	1	1	1	1	0	1	1	1	1	Yes
A25	Atanasova et al. [36]	1	1	1	1	1	0	1	1	1	1	Yes
A32	Atanasova et al. [27]	1	1	1	1	1	0	1	1	1	1	Yes

a: Yes = 1, No = 0, Can’t tell = 2.

The total score is calculated as the proportion of “yes”. If the score is less than 0.7, it is considered unqualified.

Part 2. Quality assessment for quantitative studies(n=15)[a]

The Quality Assessment Tool
Objectives<br><br>(1) Was the research question or objective in this paper clearly stated?<br><br>(2) Was the study population clearly specified and defined?<br><br>***Sample selection and method***<br><br>(3) Was the participation rate of eligible persons at least 50%?<br><br>(4) Sampling method: Was it representative of the population intended in the study?<br><br>(5) Was a sample size justification, power description, or variance and effect estimates provided?<br><br>(6) Was the study design appropriate for the research question?<br><br>(7) Was it a primary or secondary data source?[yes = primary, no = secondary]<br><br>(8) Does the study test a stated hypothesis?<br><br>***Design and Results***<br><br>(9) Were the independent variables clearly defined, valid, reliable, and implemented consistently across all study participants?

(10) Were the outcome measures (dependent variables) clearly defined, valid, reliable, and implemented consistently across all study participants?<br><br>(11) Were the statistical analyses performed correctly?<br><br>(12) Do the data justify the conclusions?<br><br>***Outcomes of the research***<br><br>(13) Did the researcher discuss the contribution of the study to existing knowledge or understanding？<br><br>(14) Did the researchers confirm that the study filled a gap in a new field？

ID	Study	(1)	(2)	(3)	(4)	(5)	(6)	(7)	(8)	(9)	(10)	(11)	(12)	(13)	(14)	Qualified or not?
A05	Dixon et al. [29]	1	1	1	1	1	1	1	2	1	1	1	1	0	0	Yes
A06	Lee et al. [31]	1	1	1	1	1	1	1	1	1	1	1	1	1	1	Yes
A13	Brown et al. [46]	1	1	0	1	1	1	1	2	1	1	1	1	1	1	Yes
A15	Campbell et al. [32]	1	1	0	0	1	1	1	2	1	1	1	1	1	1	Yes
A16	Liu et al.	1	1	2	1	1	1	0	1	1	1	1	1	0	1	Yes

	[33]															
A19	Yang et al. [44]	1	1	2	1	1	1	0	1	1	1	1	1	1	1	Yes
A23	Wu et al. [6]	1	1	2	1	1	1	0	1	1	1	1	1	1	1	Yes
A26	Zhang et al. [38]	1	1	1	1	1	1	1	1	1	1	1	1	1	1	Yes
A27	Petrič et al. [40]	1	1	1	1	1	1	1	2	1	1	1	1	1	1	Yes
A28	Guo et al. [3]	1	1	2	1	1	1	0	1	1	1	1	1	1	1	Yes
A29	Wu et al. [41]	1	1	1	1	1	1	1	1	1	1	1	1	1	1	Yes
A30	Wu et al. [42]	1	1	1	1	1	1	1	1	1	1	1	1	1	1	Yes
A31	Daniel et al. [37]	1	1	0	0	1	1	1	2	1	1	1	1	1	1	Yes
A33	Wu et al. [45]	1	1	2	1	1	1	0	1	1	1	1	1	1	1	Yes
A34	Guo et al. [43]	1	1	2	1	1	1	0	1	1	1	1	1	1	1	Yes

a: Yes = 1, No = 0, Not reported = 2.

The total score is calculated as the proportion of "yes". If the score is less than 0.7, it is considered unqualified.

Part 3. Quality assessment for review papers (N=5)[a]

ID	First author	Peer-reviewed or not?	Qualified or not?
A01	Ball et al. [1]	Yes	Yes
A03	McGeady et al. [15]	Yes	Yes
A04	Masters [10]	Yes	Yes
A10	Courtney [16]	No	No
A11	Gholami-Kordkheili et al. [11]	Yes	Yes

a: Review articles published in peer-reviewed journals were determined to be qualified.
